# The choice of multi-beam IMRT for whole breast radiotherapy in early-stage right breast cancer

**DOI:** 10.1186/s40064-016-2314-2

**Published:** 2016-05-28

**Authors:** Emel Haciislamoglu, Fatma Colak, Emine Canyilmaz, Ahmet Yasar Zengin, Ahmet Hakan Yilmaz, Adnan Yoney, Zumrut Bahat

**Affiliations:** Department of Radiation Oncology, Faculty of Medicine, Karadeniz Technical University, Trabzon, Turkey; Department of Radiation Oncology, Faculty of Medicine, Kanuni Research and Education Hospital, Trabzon, Turkey; Department of Nuclear Physics, Faculty of Science, Karadeniz Technical University, Trabzon, Turkey

**Keywords:** Doses to the heart and LAD, Helical tomotherapy, Multi-beam inverse planning, Right breast cancer, Volumetric arc therapy

## Abstract

The aim of this study was to identify a rational strategy for the selection of multi-beam IMRT in patients with right breast cancer through the comparison of dosimetric parameters of the planning target volume (PTV) and organs at risk (OARs) using five different radiotherapy modalities. This was a retrospective study using computed tomography scans from ten patients with early-stage right breast cancer who had been treated previously. Three dimensional conformal radiotherapy (3DCRT), forward-planned IMRT (for-IMRT), inverse-planned IMRT (inv-IMRT), helical tomotherapy (HT), and volumetric-modulated arc therapy (VMAT) were planned for each patient. The plans were compared according to dose–volume histogram analysis. The most significant impact of inverse-planned multi-beam modalities for right breast cancer was the reduction of D_max_, D_mean_, V_53.5_ and prescribed dose volume (cc) outside of the PTV (breast) (OB-V_50_) of the PTV. HT decreased the ipsilateral OAR volumes receiving higher doses. In exchange, HT also increased the volumes receiving low doses, which is known to lead to an increased rate of radiation-induced secondary malignancies. The heart, LAD, and contralateral doses for 3DCRT and for-IMRT were significantly lower than those for inv-IMRT, HT, and VMAT. In addition, inv-IMRT demonstrated an increase in exposed volume of heart, LAD, ipsilateral lung, and contralateral lung compared with those parameters for HT or VMAT. Although it is known to reduce cardiac toxicity with breath hold technique in left sided breast cancer, similarly it is possible for 3DCRT and for-IMRT techniques in right sided breast cancer even in free breathing.

## Background

Breast cancer is the most common cancer in females worldwide, and radiotherapy (RT) is a vital component in breast cancer management (Overgaard et al. 1997; Ragaz et al. 1997). There are various methods to employ radiotherapy for breast cancer. For example, three dimensional conformal radiotherapy (3DCRT) using wedged tangential fields after breast-conserving surgery improves disease control and breast-cancer related survival. 3DCRT reduces normal tissue doses and increases conformity to target volume.

With the advent of advanced sophisticated treatment planning software and multi-leaf collimators (MLC), intensity-modulated radiotherapy (IMRT) is becoming increasingly popular and widely used for the treatment of breast carcinoma. IMRT is thought to result in a preferred dose distribution compared to 3DCRT after conservative surgery or mastectomy (Cozzi et al. 2005; Fong et al. 2009; Johansen et al. 2009; Vatanen et al. 2009). The IMRT allows the user to modulate the intensity of each radiation beam, so each field may have one or many areas of high intensity radiation and any number of lower intensity areas within the same field.

The result is greater control of the dose distribution within the target area. To date, several publications have reported that IMRT, including forward-planned field-in-field IMRT (for-IMRT), inverse-planned IMRT (inv-IMRT), and helical tomotherapy (HT), results in a preferred dose distribution compared to 3DCRT for the RT of breast cancer (Barnett et al. 2009; Zhang and Zheng 2011). However, there have been conflicting reports on the performance of for-IMRT, inv-IMRT, and HT, and it is unclear which of these techniques is superior (Caudrelier et al. 2009; Moon et al. 2009; Coon et al. 2010; Gauer et al. 2010; Hijal et al. 2010; Qiu et al. 2013). Moreover, most of these studies have examined left-sided breast irradiation, and relatively little literature exists evaluating right-sided whole breast irradiation.

Dosimetric benefit of IMRT has been established for left breast cancer (Barnett et al. 2009; Zhang and Zheng 2011; Haciislamoglu et al. 2015). However, IMRT is not routinely employed for right breast cancer. In this study, we retrospectively analyzed the dosimetric and technical differences among 3DCRT, for-IMRT, inv-IMRT, HT, and volumetric-modulated arc therapy (VMAT) in 10 patients with right-sided breast cancer who had received conservative surgery and were previously irradiated with 3DCRT or for-IMRT. We quantitatively compared the quality of treatment plans according to dose uniformity and conformity in breast volume. We also examined the dose to the surrounding normal tissues of lungs, heart, left anterior descending artery (LAD) and contralateral breast.

## Methods

### Patient selection, positioning, and computed tomography scanning

Ethics committee approval was received for this study from the ethics committee of Karadeniz Technical University, Farabi Hospital. This study was conducted using treatment plans done on the computed tomography (CT) simulation data sets (5 mm slice thickness) of 10 consecutive right-sided breast cancer patients who had been previously treated with T1N0 carcinoma at our clinic. Patients were placed in the supine position on a breast board with ipsilateral arm raised above the head. Patients were scanned with coverage of all the inferior, superior, and lateral borders of the whole breast and critical organs. After the planning CT was done, the Digital Imaging and Communication in Medicine (DICOM) images were transferred to the Eclipse (version 10, Varian Medical Systems) treatment planning system (TPS).

### Target and organ at risk delineation

Auto contouring was used on the body and both lungs. The delineation of target and critical structures (heart, LAD, and the contralateral breast) for all patients was determined by a single radiation oncologist with extensive experiences in the treatment of breast cancer to prevent personal contouring differences that could skew the study results. The clinical target volume (CTV) was defined as the glandular breast tissue apparent on the CT scan. The PTV was determined as the CTV retracted 5 mm from the skin surface (Schubert et al. 2011). The purpose of this retraction was to account for dose buildup during dose calculation. For this reason, the PTV was used for target coverage comparisons. A new planning volume, the PTV-IMRT, was defined to facilitate inv-IMRT planning optimization, and this planning volume was created with a margin of 5 mm on the breast PTV to assure coverage of the treatment area (Mayo et al. 2005; Schubert et al. 2011). The organs at risk (OARs) included the ipsilateral lung (IL), the contralateral lung (CL), the heart, LAD, and the contralateral breast (CB). The LAD was contoured by the reference of RTOG organ at risk atlas. The CTV, PTV, and OARs were generated in accordance with the Radiation Therapy Oncology Group (RTOG) 0319 protocol (D’Arienzo et al. 2012).

An additional structure, which was specified as Body-PTV, was created to evaluate the effects of low doses on the body excluding the PTV. With the use of anatomic references, Body-PTV was defined superiorly at the T1 thoracic vertebrae level and inferiorly at the L1 lumbar vertebrae level. Boolean operations were used to construct a modified body volume that excluded breast tissue with a 1-cm margin. CT images of each patient with complete target and organ structure information were transferred into the tomotherapy planning systems.

### Treatment planning details and dose prescription

All 3DCRT, for-IMRT, inv-IMRT and VMAT plans were designed on the Eclipse planning system (version 10), while HT planning was conducted on the Hi-Art (version 4.1.2) treatment planning system. All treatment plans were generated with 6 MV (Mega voltage) photon beams to maintain the comparison between the five treatment techniques. The Pencil Beam Convolution algorithm was used to calculate the optimal dose for all plans. The prescribed dose to the PTV was 50 Gy in 2 Gy daily fractions. The treatment plans were optimized to meet the planning objectives and to achieve the prescribed dose delivery for > 90 % of the prescribed isodose (45 Gy) to encompass greater than 95 % of the PTV volume. The dose–volume constraints used for the targets and critical structures are listed in Table 1. These constraints were based on experience in our clinic and kept the same for all plans.

**Table 1:** Dose–volume constraints for target and organs at risk (OARs)

Target or OAR	Goal or constraint dose (%)
Planning target volume (PTV)	45 or 47.5 Gy
Heart	V_20_ < 10 %
	V_30_ < 3 %
LAD	D_max_ < 15 Gy
	D_mean_ < 5 Gy
Ipsilateral lung	V_20_ < 20 %
	V_50_ = 0
Contralateral lung	V_5_ < 20 %
	V_10_ = 0
Contralateral breast	D_mean_ < 4 Gy
	D_max_ < 10 Gy

### Forward-planned modalities (3DCRT and for-IMRT)

For the 3DCRT technique, the beam arrangement consisted of two parallel opposing tangential beams to ensure the best possible coverage of the breast tissue and to minimize the dose to the adjacent critical structures. The “isocenter” of the treatment machine was positioned at the center point of the midline that joins two parallel opposing fields. Dynamic wedges were then added to both tangential beams in order to improve the dose uniformity to the PTV. The for-IMRT technique used two tangential fields with the same beam angles and same isocenter point as that used for 3DCRT, and 2- to 4-segment field-in-field modulation was used to homogenize the breast dose for each field.

### Inverse-planned modalities (inv-IMRT, HT, and VMAT)

For inv-IMRT, nine beams with different gantry angles were defined and optimized to meet the requirements established for irradiating the PTV and sparing the OARs. The lateral and medial gantry angles were the same as those used for 3DCRT and for-IMRT, and the other seven fields were placed between these fields at equal intervals. The “isocenter” of the treatment machine was positioned at the same point as that used for the 3DCRT and for-IMRT plans. For HT, the parameters affecting dose conformity and treatment times are the field width (FW), pitch, and modulation factor (MF). This study utilized a 2.5-cm FW, a pitch of 0.287 and an MF of 2.5. The virtual structure (constraint heart and LAD) for dose constraint was contoured for each patient to increase the dose conformity of the PTV and to decrease the dose to the lungs, LAD, and heart. Directional or partial blocking was applied to the virtual structure and partial blocking was applied to the CB. Critical structures and virtual structure volume dose constraints were set in the optimization procedure. For the VMAT plan, a partial arc arrangement was selected in order to minimize dose to the normal structures. The first step was to determine the partial arc range based on the PTV location. Starting and ending beam angles of the arc were 10 degrees posterior to the tangential fields used for 3DCRT and for-IMRT.

### Plan comparisons

Dosimetric comparisons of the plans were performed based on the following parameters extracted from the dose–volume histogram (DVH): D_max_ and D_mean_ of PTV (D_max_ = dose received by 0.1 % volume of PTV, D_mean_ = mean dose of PTV), V_53.5_ (relative volume of breast PTV receiving 107 % of the prescription dose), IB-V_50Gy_ (prescribed dose volume (cc) inside of the PTV), OB-V_50Gy_ (prescribed dose volume (cc) outside of the PTV), conformation number (CN) and homogeneity index (HI). Additionally, dose and volume parameters of the OARs were examined. For all treatment plans, the DVH of the normal tissue-sparing (Body-PTV) and monitor unit (MU) settings required for each plan were calculated and compared.

HI was calculated from the following formula:

$${\text{HI}} = \left( {{\text{D}}_{2} - {\text{D}}_{98} /{\text{D}}_{50} } \right) \times 100\;\%$$

where D_98_ = the corresponding dose for 98 % of the target volume measured on the DVH, and D_2_ = the corresponding dose for 2 % of the volume on the DVH. The HI formula shows that lower HI values indicate a more homogeneous target dose.

The CN value was calculated as previously proposed by Van’t Riet et al. (1997). This CN simultaneously takes into account irradiation of the target volume and irradiation of the healthy tissues. CN is calculated from the following formula:

$${\text{CN}} = \left( {{\text{TVRI}}/{\text{TV}}} \right)\left( {{\text{TVRI}}/{\text{VRI}}} \right)$$

where TVRI = target volume covered by the reference isodose (95 % of the prescribed dose), TV = target volume, and VRI = volume of the reference isodose. The CN ranges from 0 to 1, where 1 is the ideal value.

### Statistical analysis

Statistical analysis was performed using SPSS software (version 13.0). Quantitative data were expressed as mean ± standard deviation (x ± SD). Multiple groups of means were compared with 1-way analysis of variance (ANOVA), after testing for equality of variance. The 2–2 comparisons were calculated using the Bonferroni test between any 2 means. A value of p ≤ 0.05 was considered statistically significant.

## Results

### Comparison of D_max_, D_mean_, CN, HI and MU for different techniques

**Fig. 1:**
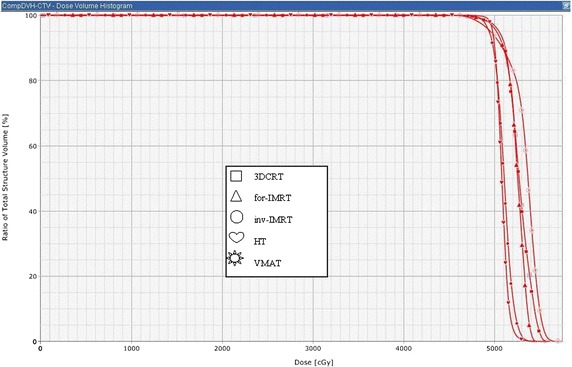
Dose–volume histograms of the five modalities for a typical patient are shown for planning target volüme

Dose–volume histogram plot of the five modalities for a typical patient for PTV is illustrated in Fig. 1. All of the evaluated modalities provided adequate coverage of the whole breast (95 % of the PTV, or ideally 47.5 Gy, but at least 45 Gy). The D_max_, D_mean_, V_53.5_ (%), IB-V_50Gy_, OB-V_50Gy_, CN, HI, and MU of all 5 techniques are summarized in Table 2.

**Table 2:** Comparision of target coverage metrics for the PTV as a function of plan modality ($\overline{x}$ ± SD)

Metric	3DCRT	for-IMRT	inv-IMRT	HT	VMAT	p-value
D_max_ (Gy)	55.94 ± 1.34	55.01 ± 1.30	54.16 ± 0.54	53.85 ± 0.66	55.08 ± 0.72	<0.001
D_mean_ (Gy)	51.86 ± 1.04	51.66 ± 1.00	51.18 ± 0.36	50.85 ± 0.22	51.35 ± 0.40	0.019
V_53.5_ (%)	20.56 ± 15.60	13.03 ± 12.80	1.85 ± 1.77	0.39 ± 0.59	10.15 ± 2.98	<0.001
IB-V_50Gy_ (cc)	765 ± 338	659 ± 423	696 ± 314	750 ± 376	717 ± 399	0.964
OB-V_50Gy_ (cc)	379 ± 323	245 ± 239	103 ± 58	76 ± 51	57 ± 29	<0.001
CN	0.58 ± 0.07	0.62 ± 0.08	0.75 ± 0.05	0.80 ± 0.05	0.73 ± 0.06	<0.001
HI	0.13 ± 0.02	0.12 ± 0.07	0.09 ± 0.01	0.06 ± 0.01	0.17 ± 0.02	<0.001
MU	231 ± 29 MU	222 ± 14 MU	1245 ± 146 MU	9284 ± 354 MU	300 ± 52 MU	<0.001

*D*_*max*_ max dose, *D*_*mean*_ mean dose, *V*_*x*_ volume (%) receiving x dose (Gy) or higher, *IB-V*_*50Gy*_ prescribed dose volume (cc) inside of the PTV, *OB-V*_*50Gy*_ prescribed dose volume (cc) outside of the PTV, $\overline{x}$ mean dose, *SD* standard deviation, *CN* conformation number, *HI* homogeneity index, *MU* monitor unit

Significant differences in D_max_ were found by 1-way ANOVA (p < 0.001). Further, the Bonferroni analysis showed that HT yielded significantly smaller values than 3DCRT, for-IMRT, and VMAT (p < 0.001, p = 0.040, p < 0.001), but no significant differences were observed between HT and inv-IMRT (p = 0.922). The D_mean_ was significantly different among the 5 techniques (p = 0.019), and Bonferroni analysis indicated that there was a significant difference in D_mean_ between HT and 3DCRT only (p = 0.002). Notably, HT showed the smallest values for D_max_ and D_mean_ among all techniques.

To evaluate the maximum doses for the PTV, the parameter V_53.5_ was used. V_53.5_ was 1.85 ± 1.77 % for inv-IMRT and 0.39 ± 0.59 % for HT, and these values were significantly different than those of other modalities. For IB-V_50Gy_, there was no significant difference between groups (p = 0.964). The inv-IMRT, HT, and VMAT plans significantly reduced OB-V_50Gy_.

The difference in HI was statistically significant among the 5 techniques (p < 0.001), and Bonferroni analysis indicated that there were no significant differences between 3DCRT and for-IMRT (p = 0.085). However, inv-IMRT and HT provided significantly superior uniformity over the other techniques (p < 0.001). VMAT plans had less homogeneity than inv-IMRT or HT. Differences in CN among the 5 techniques were statistically significant (p < 0.001), and Bonferroni analysis revealed that HT yielded superior CN compared with the other 4 types of techniques (average values closer to the ideal). Meanwhile, no significant difference was observed between 3DCRT and for-IMRT (p = 0.476) or between inv-IMRT and VMAT (p = 1.000).

In general, the inverse-planned modalities showed an increase in MU compared with forward-planned modalities. However, the MU of the VMAT plan (300 ± 52 MU) was significantly smaller than that of the inv-IMRT (1245 ± 146 MU) and the HT (9284 ± 354 MU).

### Comparison of the dosimetric parameters of OARs for different techniques

**Fig. 2:**
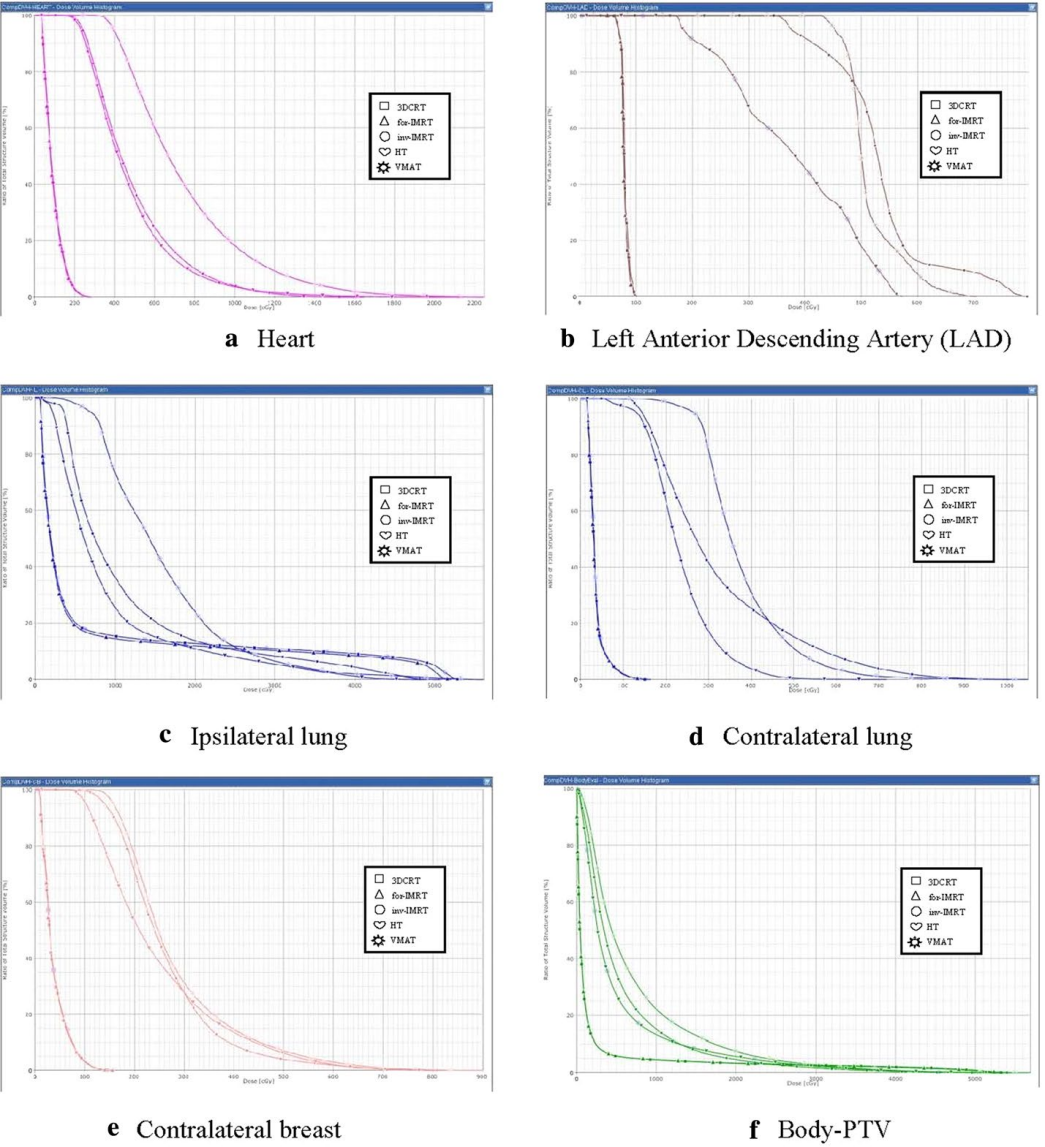
Dose–volume histograms of the five modalities for a typical patient are shown for **a** heart, **b** left anterior descending artery (LAD), **c** ipsilateral lung, **d** contralateral lung, **e** contralateral breast, and **f** Body-PTV

The DVH plots for OAR’s of the five modalities are depicted in Fig. 2. The dosimetric parameters of the OARs are listed in Table 3.

**Table 3:** Comparision of dosimetric metrics of OARs as a function of plan modality ($\overline{x}$ ± SD)

Metric	3DCRT	for-IMRT	inv-IMRT	HT	VMAT	p-value
Heart (RB)						
D_max_ (Gy)	2.73 ± 0.56	2.73 ± 0.52	17.80 ± 5.46	15.65 ± 3.38	12.87 ± 2.15	<0.001
D_mean_ (Gy)	0.95 ± 0.17	0.93 ± 0.15	5.10 ± 1.20	3.74 ± 0.92	4.99 ± 0.76	<0.001
V_5_ (%)	0.0 ± 0.0	0.0 ± 0.0	39.50 ± 19.02	27.60 ± 15.16	38.00 ± 12.10	<0.001
V_10_ (%)	0.0 ± 0.0	0.0 ± 0.0	7.00 ± 4.26	3.50 ± 3.42	3.90 ± 2.95	<0.001
LAD (RB)						
D_max_ (Gy)	1.00 ± 0.13	1.01 ± 0.16	8.10 ± 3.42	6.54 ± 1.85	4.63 ± 0.83	<0.001
D_mean_ (Gy)	0.85 ± 0.20	0.81 ± 0.10	5.05 ± 1.56	3.49 ± 1.20	3.71 ± 0.67	<0.001
Ipsilateral lung						
D_max_ (Gy)	51.50 ± 1.82	50.56 ± 1.34	45.96 ± 2.52	43.13 ± 4.32	44.26 ± 3.15	<0.001
D_mean_ (Gy)	7.66 ± 2.50	7.49 ± 2.44	12.18 ± 3.21	4.93 ± 1.45	11.71 ± 3.47	<0.001
V_5_ (%)	19.80 ± 7.12	12.93 ± 4.70	79.92 ± 13.02	49.40 ± 15.51	76.38 ± 20.37	<0.001
V_10_ (%)	15.21 ± 5.05	15.22 ± 5.22	44.26 ± 20.71	20.92 ± 6.58	40.30 ± 16.07	<0.001
V_20_ (%)	12.99 ± 4.60	12.93 ± 4.70	15.64 ± 7.37	6.91 ± 2.03	14.69 ± 5.52	0.005
Contralateral lung						
D_max_ (Gy)	1.52 ± 0.38	1.58 ± 0.37	11.67 ± 3.30	11.49 ± 4.22	6.25 ± 1.30	<0.001
D_mean_ (Gy)	0.35 ± 0.08	0.35 ± 0.08	3.69 ± 1.13	2.46 ± 0.96	2.50 ± 0.57	<0.001
V_5_ (%)	0.0 ± 0.0	0.0 ± 0.0	25.75 ± 18.43	14.16 ± 11.82	2.46 ± 4.55	<0.001
V_10_ (%)	0.0 ± 0.0	0.0 ± 0.0	1.73 ± 2.27	1.41 ± 2.43	0.0 ± 0.0	0.016
Contralateral breast						
D_max_ (Gy)	2.53 ± 0.91	2.66 ± 0.99	7.90 ± 2.76	9.68 ± 1.88	9.58 ± 2.99	<0.001
D_mean_ (Gy)	0.54 ± 0.14	0.53 ± 0.14	2.69 ± 0.78	2.96 ± 0.57	2.19 ± 0.49	<0.001
V_3_ (%)	0.16 ± 0.31	0.17 ± 0.36	27.87 ± 15.98	48.72 ± 15.42	17.80 ± 8.79	<0.001
V_5_ (%)	0.0 ± 0.0	0.0 ± 0.0	6.25 ± 6.98	14.54 ± 7.06	3.84 ± 3.87	<0.001
Body-PTV						
V_3_ (%)	7.81 ± 3.35	7.86 ± 3.05	51.98 ± 11.50	39.30 ± 10.30	40.77 ± 9.68	<0.001
V_5_ (%)	5.76 ± 2.38	5.73 ± 2.23	34.57 ± 9.10	27.19 ± 8.31	25.79 ± 7.98	<0.001
V_10_ (%)	4.53 ± 1.96	4.52 ± 1.88	15.32 ± 4.46	13.86 ± 4.66	11.69 ± 4.11	<0.001
V_20_ (%)	3.45 ± 1.62	3.41 ± 1.59	4.91 ± 1.68	5.95 ± 2.24	4.67 ± 1.91	0.016
V_30_ (%)	2.80 ± 1.43	2.77 ± 1.42	1.92 ± 0.88	2.39 ± 1.07	1.66 ± 0.85	0.124

*D*_*max*_ max dose, *D*_*mean*_ mean dose, *V*_*x*_ volume (%) receiving x dose (Gy) or higher

The 3DCRT and for-IMRT plans achieved similar sparing of the heart and LAD. Between forward and inverse-planned modalities, significant differences were uncovered between the D_max_, D_mean_, and V_5_ of the heart, and the D_max_ and D_mean_ of LAD. These results are summarized in Table 4. for-IMRT and 3DCRT spared a greater heart volume than inverse-planned modalities (V_5_ = V_10_ = 0 for 3DCRT and for-IMRT), and the differences between HT, inv-IMRT, and VMAT were not significant (Table 4). HT showed superior sparing of the heart compared with inv-IMRT and VMAT; however, no significant differences were found between inv-IMRT, VMAT, and HT.

**Table 4:** Estimated *p*-values for compared treatment modalities for heart, LAD and contralateral breast

Metric	A versus B	A versus C	A versus D	A versus E	B versus C	B versus D	B versus E	C versus D	C versus E	D versus E
Heart (RB)										
D_max_	NS	<0.001	<0.001	<0.001	<0.001	<0.001	<0.001	NS	0.007	0.013
D_mean_	NS	<0.001	<0.001	<0.001	<0.001	<0.001	<0.001	0.002	NS	0.007
V_5_ (%)	NS	<0.001	<0.001	<0.001	<0.001	<0.001	<0.001	NS	NS	NS
V10 (%)	NS	<0.001	NS	0.030	<0.001	NS	0.030	NS	NS	NS
LAD (RB)										
Dmax	NS	<0.001	<0.001	<0.001	<0.001	<0.001	<0.001	NS	0.002	0.016
Dmean	NS	<0.001	<0.001	<0.001	<0.001	<0.001	<0.001	0.022	0.026	NS
CB (RB)										
V_3_ (%)	NS	<0.001	<0.001	0.006	<0.001	<0.001	0.006	0.001	NS	<0.001
V_5_ (%)	NS	NS	<0.001	NS	NS	<0.001	NS	0.003	NS	<0.001

A, three-dimensional conformal radiotherapy (3DCRT); B, forward-planned intensity-modulated radiotherapy (for-IMRT); C, inverse-planned intensity-modulated radiotherapy (inv-IMRT); D, helical tomotherapy (HT) and E, volumetric-modulated arc therapy (VMAT); RB, right breast, CB, contralateral breast and NS, no significant statistical differences

The D_max_ of the LAD was significantly different between the 5 techniques (p < 0.001). Further statistical analysis revealed that VMAT yielded smaller D_max_ values than inv-IMRT and HT, and no significant difference between inv-IMRT and HT was observed. Inverse-planned modalities resulted in the lowest maximum dose and the largest low-dose volume in the ipsilateral lung compared to 3DCRT and for-IMRT. The ipsilateral lung mean dose was higher for inv-IMRT and VMAT than for 3DCRT and for-IMRT, but this dose was significantly lower for HT than for 3DCRT or for-IMRT.

Contralateral lung mean and maximum doses were higher for all inverse-planned modalities. The relative volume of contralateral breast that received the mean, maximum, and low doses was significantly lower for forward planned tangential fields (FPTF) plans than for inverse planned multi-beam (IPMB) modalities. HT demonstrated an increase in the exposed volume of contralateral breast V_3_ and V_5_ values compared with inv-IMRT and VMAT. The three IPMB techniques examined exhibited statistically significant differences in Body-PTV compared to the FPTF modalities. inv-IMRT involved the greatest spread of mainly low doses to the Body-PVT (verified by V_3_ and V_5_ in Table 3), whereas 3DCRT and for-IMRT had the lowest values for Body-PTV (expect for the fraction of Body-PTV volume receiving >30 Gy).

## Discussion

Thousands of women who are diagnosed with breast cancer each year receive breast-conserving surgery followed by adjuvant radiation therapy. Over the past decade, there has been a rapid rise in the application of advanced radiation delivery technologies and clinical irradiation patterns have shifted from conventional 2D therapy to a more developed 3D therapy based on CT for the curative management of breast cancer (Haffty et al. 2008). Breast irradiation has been shown to decrease the risk of local recurrence after breast-conserving surgery with few adverse effects (Fisher et al. 2002). One of the most concerning complications of breast radiotherapy is cardiotoxicity from radiation to the heart. Early studies showed decreased left ventricular function in breast cancer patients treated with radiation (Wehr et al. 1982). Therefore, several broad categories of techniques to reduce cardiac radiation doses were developed. These techniques include breath holding techniques, prone positioning, and two-tangential or multi-beam IMRT. However, this manuscript focused solely on beam arrangement to decrease the radiation to the heart, LAD, and contralateral breast for women receiving radiation to the right breast.

A very recent study on “Risk of Ischemic Heart Diseases in Women after Radiotherapy for Breast Cancer” by Sarah CD et al. suggests that the exposure of heart to ionizing radiations during radiotherapy for breast cancer increases the subsequent rate of ischemic heart diseases. The increase is proportional to the mean dose to heart, begins within few years after exposure, and continues at least for 20 years. Woman with preexisting cardiac risk factors have greater absolute increase in risk from radiotherapy than other women. Also, the rate of major coronary events increased linearly with the mean dose to the heart by 7.4 % per Gray, with no apparent threshold. The risk starts within 5 years after radiotherapy and continuous up to the third decade after radiotherapy (Darby et al. 2013). In our study the mean dose that heart receives were about 1 Gy for both 3DCRT and for-IMRT. The heart doses for inv-IMRT, HT, VMAT were 5.10, 3.74 and 4.99 Gy respectively. Therefore heart dose should be taken into consideration while planning the appropriate technic for right breast irradiation.

There are several published studies on the various techniques to decrease heart irradiation in women treated for left-sided breast cancer (Mayo et al. 2005; Schubert et al. 2011; Haciislamoglu et al. 2015), but relatively little published experience exists for the right breast. The objective of this study was to compare the dosimetric characteristics of IPMB (inv-IMRT, HT and VMAT) and FPTF (3DCRT and for-IMRT) techniques and to evaluate the characteristics of each modality when applied to the whole right breast in the early stage of the breast cancer.

In this study, IPMB modalities performed better than FPTF techniques for right-sided breast cancer in several respects. First, IPMB modalities provided superior dose homogeneity and conformity of PTV (except for HI of VMAT). Additionally, IPMB modalities showed a reduction in D_max_, D_mean_ and V_53.5_. The reduction of maximum breast and skin doses harbors clinical relevance because it relates to acute skin toxicity, long-term fibrosis and adverse cosmetic outcomes. Meanwhile, IPMB modalities increase the contralateral OAR volumes receiving exposure. Contrary to the left breast, contralateral OARs include the heart and LAD on right breast. Prolonged follow-up showed an increased RT-induced risk of cardiac events and secondary lung and breast cancer in long-term survivors (Darby et al. 2011; Henson et al. 2013). Moreover, according to the Ashraf et al. recent study on “Comparative Study of 3DCRT versus IMRT in Post lumpectomy Early Stage Breast Cancer Patients”, 3DCRT reduces the risk of radiation-induced heart diseases by a factor of about 9.62 in right-sided breast diseases compared to IMRT, and by a factor of 1.27 in left-sided breast diseases (Ashraf et al. 2014).

When we consider IMRT as a replacement for conventional treatment, two factors must be taken into account: (1) more monitor units are used, which results in a larger total-body radiation dose and (2) more fields are used, which results in a larger volume of normal tissue exposed to lower radiation doses (Hall and Wuu 2003; Kry et al. 2005). Some machines leak a little more than others, but the overall conclusion is that IMRT may approximately double the induced-cancer rate compared with conventional treatment. Compared with three dimensional conformal RT (3D-CRT), IMRT may double the incidence of solid cancers in long-term survivors because of a combination of the increase in monitor units and the changed dose distribution. The importance of a larger volume of normal tissue exposed to lower radiation doses depends on the shape of the dose–response relationship for radiation-induced carcinogenesis. Therefore, strategies for OARs, while maintaining adequate dose coverage of the target, are warranted. In our study, compared to IPMB modalities, FPTF modalities showed smaller exposed volumes of the heart, LAD, ipsilateral lung, contralateral lung, and breast.

Among IPMB modalities performed with respect to conservative surgery, HT provides better dose homogeneity and conformity of PTV, decreases the heart and LAD volumes receiving higher and lower doses (except for D_max_ of LAD), and has the distinctive advantage of target coverage compared with the inv-IMRT and VMAT. In our study of HT, the virtual structure for the dose constraint was contoured for each patient to decrease the dose to the heart and LAD, and this virtual structure volume was optimized similarly to that done for the left breast. During optimization, the dose to the virtual structure was reduced by setting higher dose constraints to reduce the doses to the heart and LAD. On the contrary, HT decreases the OAR volumes receiving higher doses with an increase in the volumes receiving low doses. These effects are probably due to more scattered irradiation, which is known to lead to an increased rate of radiation-induced coronary heart disease and secondary malignancies, such as cancer of the contralateral breast. HelicalTomotherapy technique where a continuous helical beam trajectory was used for this study. The TomoDirect treatment delivery technique uses two or more fixed gantry angles, as distinct from the Helical Tomotherapy. TomoDirect delivery is able to create a dose distribution very similar to that resulting from fixed-gantry 3DCRT and for-IMRT, wedged, or compensated beam techniques used with a conventional linear accelerator. Further work should be carried out to investigate the potential benefits of TomoDirect beam configurations other than simple opposed tangents.

This study suggests that IPMB reduced the maximum dose to the target volume over that achieved by FPTF; the maximum doses to ipsilateral OARs were reduced as well. However, 3DCRT and for-IMRT techniques were superior in terms of minimizing the dose to normal tissues, the dose to the heart, LAD, contralateral breast, and treatment time. Particularly, consequences of heart and LAD doses would have to be weighed against the benefits of reducing high doses on individual patient selection basis for the right breast. Therefore, the dosimetric superiority of IPMB modalities over FPTF modalities for right-sided breast cancer remains questionable, and patient-specific conditions should be included in the evaluation of which right-sided breast cancer patients may benefit from multi-beam inverse-planned IMRT.

As a result, the quality of the treatment plan depends on many factors. In general, for every patient, there is an optimum plan that treats the breast tissue while sparing the organs at risk. However, the technique one uses could vary depending on patient geometry or the technology available in the radiotherapy center, including the available treatment planning systems, beam energy, TPS algorithm, and the skills of the planner (Lu 2013; Rana 2014).

## Conclusions

For patients with right-sided breast cancer, IPMB modalities demonstrated clear advantage over 3DCRT and for-IMRT in target coverage and conformity. IPMB modalities decrease the ipsilateral OAR volumes receiving high doses, but increase the volumes receiving low doses. Additionally, IPBM modalities result in an increase in D_max_, D_mean_, and volumes receiving low doses for contralateral critical organs. The breath-hold technique resulted in a significant reduction in radiation dose to the heart and LAD compared with an free breathing technique for left breast radiotherapy. In our study we also found that 3DCRT and for-IMRT showed dosimetric benefit in right sided breast cancer. This dosimetric benefit was both useful for LAD and heart doses. The anatomic localization of the heart may cause this benefit even in the absence of breath hold technics. We infer from this study that treatment technique selection is an important factor in whole breast irradiation for both left and right breast cancers.

The IMRT plans contribute a modestly higher dose to adjacent healthy tissues. The main concern of with the healthy soft tissue dose increases of such magnitude is an increased risk of late secondary malignancy. Further, clinic studies designed to clarify uncertain as side effects and second primary tumors for right-sided breast irradiation should be performed in future.

## Abbreviations

PTV: planning target volume
OARs: organs at risk
OB-V_50_: prescribed dose volume (cc) outside of the PTV (breast)
3DCRT: three dimensional conformal radiotherapy
for-IMRT: forward-planned IMRT
inv-IMRT: inverse-planned IMRT
HT: helical tomotherapy
VMAT: volumetric-modulated arc therapy
IPMB: inverse-planned multi-beam
RT: radiotherapy
LAD: left anterior descending artery
CT: computed tomography
DICOM: Digital Imaging and Communication in Medicine
TPS: treatment planning system
CTV: clinical target volume
CN: conformation number
HI: homogeneity index
MU: monitor unit
IL: ipsilateral lung
CL: contralateral lung
CB: contralateral breast
